# Pregestational and Gestational Exposure to Wood Smoke-Derived PM_2.5_ Is Associated with Structural Remodeling of the Maternal Aortic Arch and Hemodynamic Changes During Pregnancy in Rats

**DOI:** 10.3390/toxics14060489

**Published:** 2026-06-03

**Authors:** Paulo Salinas, Francisca Villarroel, Mónica Conforti, Andrea González-Rojas, Eva Rojas, Aliro Maulén

**Affiliations:** 1 Laboratory of Animal and Experimental Morphology, Institute of Biology, Faculty of Sciences, Pontificia Universidad Católica de Valparaíso, Avenida Universidad 330, Valparaíso 2373223, Chile; 2 Escuela de Kinesiología, Pontificia Universidad Católica de Valparaíso, Avenida Universidad 330, Valparaíso 2373223, Chile; 3 Laboratorio de Ciencias Biomédicas Aplicadas a la Kinesiología (CIBAK), Escuela de Kinesiología, Pontificia Universidad Católica de Valparaíso, Avenida Universidad 330, Valparaíso 2373223, Chile; 4 Morphohistology Unit, School of Sciences, Universidad de Viña del Mar, Viña del Mar 2520000, Chile

**Keywords:** PM2.5, wood smoke, biomass combustion, aortic arch, vascular remodeling, gestational hemodynamics, pregestational exposure, arterial wall morphometry, pregnancy, rat model

## Abstract

Chronic exposure to fine particulate matter (PM_2.5_) derived from wood combustion represents a major environmental health burden, particularly during pregnancy. However, the impact of pregestational and gestational (PM_2.5_) exposure on the maternal great vasculature remains largely unexplored. This study evaluates the effects of wood smoke-derived (PM_2.5_) on the structural architecture of the maternal aortic arch and associated hemodynamic changes during pregnancy in second-generation Sprague–Dawley rats. Animals were allocated into four groups (*n* = 12) according to filtered (FA) or non-filtered air (NFA) exposure during pregestational and gestational periods: FA/FA, FA/NFA, NFA/FA, and NFA/NFA. Morphometric analysis revealed significant reductions in tunica media (*p* = 0.0251) and adventitia thickness (*p* = 0.0014) in exposed groups, without changes in integrated optical density, suggesting alterations in elastic matrix organization without evidence of net mass loss. Histological analysis supported exposure-dependent structural heterogeneity, including elastic lamellae fragmentation and extracellular matrix disorganization. Each exposed group exhibited a distinct systolic blood pressure trajectory across gestation, with FA/NFA reaching the highest values at day 18 (151.0 ± 17.0 mmHg) and NFA/FA displaying sustained elevations despite gestational low-exposure conditions. Principal component analysis (49.2% explained variance) revealed a structured multivariate distribution of vascular and hemodynamic variables across exposure conditions, consistent with an exposure-window-dependent pattern. These findings suggest that (PM_2.5_) exposure is associated with coordinated structural and hemodynamic changes in the aortic arch and support the hypothesis that the pregestational period may represent a window of increased susceptibility.

## 1. Introduction

Chronic exposure to fine particulate matter (PM_2.5_) constitutes one of the leading modifiable environmental risk factors globally, affecting more than 90% of the world’s population at levels exceeding World Health Organization (WHO) recommended limits [1,2], with health effects comparable to those of tobacco smoking [1] and contributing to over six million premature deaths annually from cardiovascular and respiratory causes [3,4]. In Chile, this burden is particularly acute in southern cities such as Temuco, where residential wood combustion accounts for more than 84% of wintertime PM_2.5_ emissions [5]; episodic concentrations exceed 370 μg/m^3^ [6,7], placing this city among the highest biomass combustion pollution environments in Latin America [8,9]. During pregnancy, maternal PM_2.5_ exposure has been associated with low birth weight, preterm birth, gestational hypertension, gestational diabetes, and preeclampsia [10,11], with the periconceptional period identified as an independent susceptibility window [12]. These effects have been linked to processes such as systemic oxidative stress, vascular inflammation, and mitochondrial dysfunction, which provide a biological framework for interpreting the systemic consequences of PM_2.5_ exposure during gestation [13,14,15]. Nevertheless, the available evidence has focused predominantly on fetal, placental, and neonatal outcomes; the impact of PM_2.5_ on the maternal large-caliber vasculature remains largely uncharacterized.

The aortic arch is a key structural determinant of gestational hemodynamic regulation. During pregnancy, it accommodates increases in cardiac output exceeding 40%, reductions in peripheral vascular resistance, and expansion of circulating volume through adaptive wall remodeling involving smooth muscle cell hypertrophy and extracellular matrix reorganization [16,17,18]. Under PM_2.5_ exposure conditions, smooth muscle cell phenotypic switching and extracellular matrix accumulation have been reported [19,20,21,22], suggesting a potential disruptive influence on gestational arterial remodeling. Evidence from our group using a multigenerational model of controlled wood smoke PM_2.5_ exposure has documented a convergent systemic maternal phenotype characterized by placental hypoxia, altered nutrient transport, and structural changes in reproductive and extra-reproductive organs, collectively suggesting a pattern of structural and functional alterations potentially associated with oxidative stress and extracellular matrix remodeling [19,20,23,24,25,26]. Despite this emerging evidence, the aortic arch has not been evaluated as a direct target organ of PM_2.5_ during pregnancy.

This gap can be summarized in three key unresolved dimensions: (i) the absence of studies addressing large-caliber vasculature under gestational conditions, given that the existing literature on PM_2.5_ and arterial structure has focused exclusively on non-pregnant populations and on documenting arterial stiffness [27,28,29], central hemodynamic dysfunction [30], and aortic structural changes in atherosclerotic models [31,32] without addressing a vascular bed undergoing active remodeling to sustain uteroplacental perfusion [17,33]; (ii) the limited understanding of whether PM_2.5_-associated structural remodeling mechanisms, including oxidative stress in the perivascular adipose tissue and disorganization of collagen and smooth muscle fibers [31,34], are amplified when the extracellular matrix and smooth muscle cell population are already operating under conditions of heightened phenotypic plasticity [18]; and (iii) the unexplored role of pregestational exposure as a determinant of vascular programming, whereby structural conditioning of the arterial wall prior to conception may modulate gestational adaptive capacity independently of active exposure [12,35].

Against this background, we pose the following research question: does pregestational and gestational exposure to wood smoke-derived PM_2.5_ induce changes in the histological and morphometric architecture of the aortic arch in pregnant rats, and are these modifications associated with variations in blood pressure during gestation? The objective of this study is to evaluate the impact of such exposure on the structural organization of the maternal aortic wall and to examine its association with gestational hemodynamic changes, integrating morphometric and physiological variables through univariate, bivariate, and multivariate analytical approaches. We hypothesize that pregestational and gestational exposure to wood smoke-derived PM_2.5_ is associated with differential structural remodeling of the maternal aortic wall and distinct gestational hemodynamic profiles, with the timing of exposure modulating the magnitude and pattern of these integrated vascular responses.

## 2. Materials and Methods

### 2.1. Location, Air Pollution Exposure and Filtration System

The study was conducted in Temuco, La Araucanía Region, Chile (Supplementary Document S1; 38°44′59.4″ S, 72°37′07.8″ W), an urban area characterized by residential wood burning as the main source of both heating and airborne particulate matter. No major industrial emission sources were identified in the study area. The effects of wood smoke-derived PM_2.5_ on aortic morphology and maternal blood pressure were evaluated using a controlled environmental exposure model based on parallel filtered-air and non-filtered-air chambers. This system allowed environmentally relevant differences in particulate matter concentration to be reproduced under controlled conditions [36]. The study was conducted during the austral winter, from 9 July to 30 September 2021, a period associated with peak ambient PM_2.5_ concentrations in the region.

**Figure 1:**
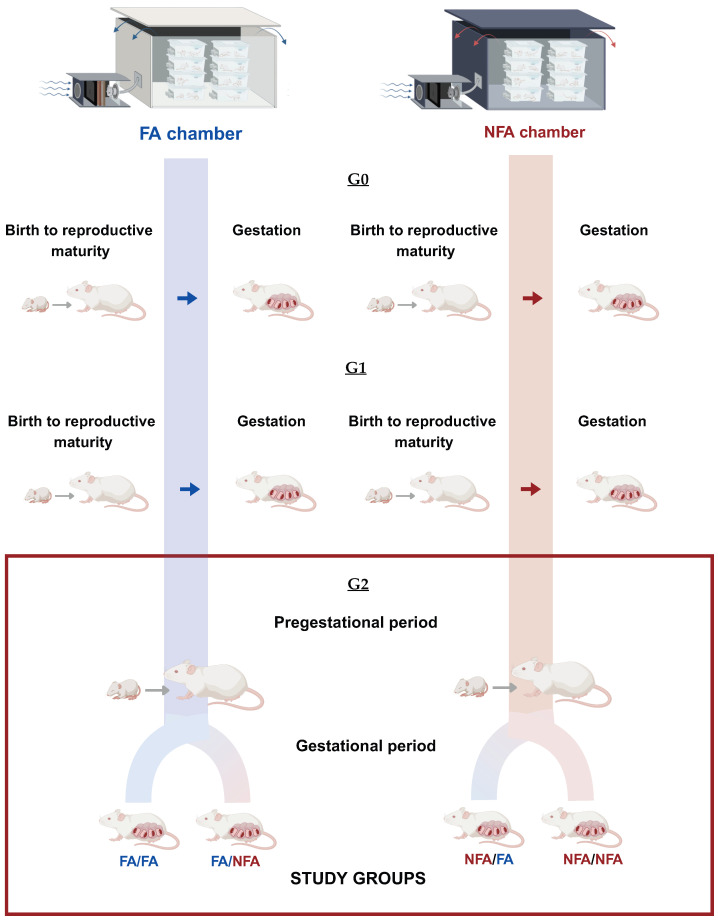
Exposure, filtration, and reproduction in rats. Filtered air chamber (FA) designed to supply filtered air and reduce PM_2.5_ exposure. Non-filtered air chamber (NFA) allowing for exposure to ambient air containing particulate matter. G2 experimental design illustrating multigenerational exposure and experimental groups (FA/FA, FA/NFA, NFA/FA, NFA/NFA).

The exposure system consisted of two identical chambers (2.1 m × 2.0 m × 2.1 m; Figure 1) positioned side by side and operated under comparable environmental conditions. Air was supplied at a constant flow rate of 20 m^3^/min through intake ports positioned 1.5 m above ground level, maintaining 15 air changes per hour. One chamber received unfiltered ambient air and was designated as the non-filtered air chamber (NFA), whereas the other incorporated a three-stage filtration system and was designated as the filtered air chamber (FA). The filtration system included metallic pre-filters for coarse particles, HEPA PH97 filters with 99.97% removal efficiency for particles larger than 0.3 μm, and a Purafil PSA 102 gas filtration unit equipped with Purafil Select media (Purafil Inc., Doraville, GA, USA).

Both chambers were maintained under standardized housing conditions, including temperature control (20–24 °C), relative humidity monitoring (40–60%), and a 12:12 h light–dark cycle. Airflow homogeneity was ensured by an internal fan (150 m^3^/h) and a wide upper exhaust outlet, minimizing spatial variability in exposure within each chamber. The chambers were located near the Las Encinas air quality monitoring station in Temuco, Chile, allowing exposure conditions to reflect representative urban wood smoke-derived PM_2.5_ concentrations during the winter period.

### 2.2. Air Analysis and Composition of PM_2.5_

Particulate matter and gaseous pollutants were monitored to characterize both chamber-specific and ambient exposure conditions throughout the experimental period. PM_2.5_ concentrations within each exposure chamber (FA; NFA) were continuously recorded over 24-h cycles using a calibrated digital particulate matter analyzer and expressed as daily mean concentrations (μg/m^3^). Ambient PM_2.5_ concentrations were obtained from the Las Encinas Monitoring Station (National Air Quality Information System) located approximately 200 m from the experimental facility, using a beta attenuation monitor (BAM 1020; Met One Instruments, Inc., Grants Pass, OR, USA) operating at 16.7 L/min and reported as hourly and 24-h averaged concentrations (μg/m^3^). In addition, PM_10_ and gaseous pollutants, including carbon monoxide (CO) and nitrogen dioxide (NO_2_), were measured to provide a comprehensive characterization of the exposure environment. Ambient CO concentrations were recorded as 8-h moving averages using ultraviolet absorption photometry (Teledyne API Model T300; Teledyne API, San Diego, CA, USA), while NO_2_ levels were determined as 24-h averages using gas-phase chemiluminescence (Thermo Scientific 42i, Thermo Fisher Scientific, Waltham, MA, USA). Because the filtration system was designed to selectively remove particulate matter without affecting gaseous components, CO and NO_2_ concentrations did not differ between FA and NFA chambers throughout the experimental period, as both chambers received equivalent gaseous pollutant loads from the same ambient air source. This differential filtration isolates PM_2.5_ as the primary exposure variable and is consistent with previous controlled exposure studies using comparable filtration configurations [36]. Accordingly, gaseous pollutants were not included in subsequent analyses, as they were not part of the primary objectives of the present study. Nevertheless, we acknowledge that gaseous co-pollutants represent a potential residual source of biological variability that cannot be fully excluded under the present experimental conditions; this constitutes a recognized limitation of ambient air exposure systems that do not incorporate gas-phase scrubbing. Instrument calibration and data acquisition followed manufacturer specifications and national air quality monitoring standards. The elemental composition of PM_2.5_ derived from wood smoke in this experimental setting has been previously characterized [24]; therefore, chemical characterization was not repeated and the present study focused on the associated biological effects.

### 2.3. Animals

This study followed a controlled experimental design in which the individual animal was considered the experimental unit. A total of 48 s-generation (G2) pregnant female Sprague–Dawley rats were included. Animals were housed in the university animal facility under controlled environmental conditions (18–26 °C, 40–60% relative humidity) and provided with a balanced diet and water ad libitum. All experimental procedures were conducted in accordance with Chilean Law 20.380 and the *Guide for the Care and Use of  Laboratory  Animals* [37] and were approved by the Bioethics Committee of Universidad de La Frontera (Approval No. 122/2020). This study was conducted and reported following the ARRIVE 2.0 guidelines. Inclusion and exclusion criteria were defined a priori. Only healthy pregnant females born and maintained exclusively within their assigned exposure condition, with confirmed gestation, normal general health status, and regular estrous cycles as determined by vaginal cytology, were included. Animals were excluded if they presented clinical signs of disease, irregular estrous cyclicity, body weight deviations outside the expected range for the strain, complications during gestation, or tissue artifacts compromising histological evaluation. Physiological monitoring included periodic assessment of estrous cycle stage by vaginal cytology, body weight measurements three times per week, and evaluation of gestational systolic blood pressure according to established protocols [38].

Maternal body weight was monitored weekly during the pregestational exposure period and every 2–3 days during gestation to confirm normal pregnancy progression. Only animals exhibiting expected gestational weight gain were included in the final sample. Detailed maternal body weight data (pregestational baseline, gestational weight gain curves, and terminal body weight at GD22) as well as reproductive outcomes (litter size, fetal weight, placental weight, crown-rump length) are reported in Salinas et al. [24]. No significant differences in maternal body weight or gestational weight gain were observed among exposure groups, confirming group comparability in terms of maternal somatic development and pregnancy-associated growth.

### 2.4. Experimental Design

The present study employed a controlled experimental design with longitudinal PM_2.5_ exposure across multiple developmental windows and a multigenerational structure spanning three consecutive generations (G0, G1, and G2), with the analysis focused on the G2 generation. Founder animals (G0; 10 males and 10 females) were maintained in either filtered-air (FA) or non-filtered-air (NFA) chambers (Figure 1). Their offspring (G1) remained under the same exposure conditions; upon reaching reproductive maturity they were mated within their respective groups to generate the G2 generation. The use of G2 animals was defined to capture the cumulative effects of sustained PM_2.5_ exposure across multiple developmental windows, including prenatal, postnatal, and juvenile stages, enabling the assessment of integrated biological responses resulting from continuous environmental exposure. In order to reduce variability associated with paternal environmental history, males used for breeding were maintained under standard animal facility conditions prior to mating. The G2 protocol comprised two sequential exposure phases: a pregestational period of 60 days, followed by a gestational period of 23 days. The 60-day pregestational exposure window was designed to encompass at least two complete follicular maturation cycles in rats. Female Sprague–Dawley rats reach sexual maturity at approximately 35–40 days of age [39], and the complete folliculogenesis process, from primordial follicle activation to ovulation, spans approximately 19–21 days [40]. By initiating exposure at 8 weeks of age, corresponding to sexually mature females, and continuing exposure for 60 days before mating, we ensured that the oocytes ovulated during the subsequent gestation had undergone their complete growth phase under the assigned exposure condition. This approach minimized variability associated with incomplete follicular exposure. All G2 females were sexually mature (≥8 weeks of age) at the onset of pregestational exposure, reducing potential confounding effects related to pubertal hormonal transitions or ongoing sexual maturation. Animals in the FA/NFA and NFA/FA groups were transferred between chambers at the onset of gestation under standardized handling conditions in order to minimize stress-related variability. To ensure procedural parity and minimize confounding from handling stress, all animals underwent identical handling protocols regardless of exposure group. FA/FA and NFA/NFA animals were subjected to sham chamber transfers at gestational day 0, mimicking the chamber relocation procedure (duration, restraint, transport) experienced by FA/NFA and NFA/FA groups during their actual chamber transitions. Additionally, all animals were habituated to the tail-cuff plethysmography restraint system for three consecutive days prior to baseline hemodynamic measurements, consisting of 10-min restraint periods without data acquisition to reduce acute stress responses during subsequent measurements. Allocation was determined by predefined exposure history; therefore, randomization was not applicable. Nevertheless, all environmental and experimental conditions, including housing, handling, feeding, and monitoring, were strictly standardized across groups to minimize potential sources of systematic bias. Because PM_2.5_ exposure was administered at the chamber level, all animals within a given chamber shared the same environmental conditions. Therefore, the chamber constituted the exposure unit, whereas individual animals were considered the unit of observation for morphological and physiological outcomes. This design implies a potential lack of full independence among animals within the same chamber, which represents an inherent limitation of controlled environmental exposure systems. However, environmental conditions within each chamber were stable, homogeneous, and continuously monitored, allowing for the assessment of individual-level biological responses under consistent exposure scenarios. Accordingly, the results were interpreted considering the chamber as a potential source of clustering. The sample size (n=12 per group) was defined based on previous studies employing comparable exposure systems and outcome measures together with ethical considerations aimed at minimizing animal use. Although no formal a priori power calculation was performed, the selected sample size was consistent with established experimental designs in the field and allowed for the detection of statistically significant differences in key structural outcomes. Nevertheless, this does not replace the need for future studies incorporating formal power estimations.

### 2.5. Experimental Groups

G2 pregnant females were allocated into four experimental groups (n=12 per group) according to exposure conditions during the pregestational and gestational periods: FA/FA (filtered air during both development and gestation), FA/NFA (filtered air during development and non-filtered air during gestation), NFA/FA (non-filtered air during development and filtered air during gestation), and NFA/NFA (non-filtered air during both periods). To minimize potential litter effects, a maximum of 15 animals per litter were included in each group; efforts were made to distribute animals from different litters across experimental groups, reducing potential confounding associated with shared maternal or genetic background.

### 2.6. Aortic Arch Sampling and Histological Processing

At gestational day 22 (GD22), animals were deeply anesthetized with an intraperitoneal combination of ketamine (80 mg/kg) and xylazine (10 mg/kg), followed by exsanguination as a method of euthanasia. All procedures were conducted in accordance with ARRIVE 2.0 essential guidelines. Adequate anesthesia was confirmed by the absence of reflex responses prior to tissue collection. Following euthanasia, the thoracic cavity was opened via median sternotomy. The aortic arch was selected as the sampling site for several reasons: (1) it is subject to complex hemodynamic forces, including pulsatile flow, turbulence, and high wall shear stress, making it particularly susceptible to flow-mediated remodeling; (2) it has been used as a reference site in prior studies of gestational vascular adaptation, which facilitates comparison with existing literature; and (3) practical tissue allocation constraints precluded complete longitudinal sampling of all aortic segments, as tissue was also allocated for parallel analyses not reported in this manuscript. The aortic arch was carefully dissected under a stereomicroscope (Leica M80, Leica Microsystems, Wetzlar, Germany), thereby preserving structural integrity and minimizing mechanical manipulation. The sampled segment extended from the distal ascending aorta to the proximal descending thoracic aorta, using the brachiocephalic trunk, left common carotid artery, and left subclavian artery as anatomical landmarks to ensure consistent anatomical localization across specimens. Tissue segments were oriented with the greater curvature of the arch facing upward prior to embedding, and all blocks were trimmed to yield true transverse cross-sections perpendicular to the longitudinal axis of the vessel. Section quality was verified by confirming a circular-to-elliptical luminal profile before image acquisition; sections showing oblique cutting angles (lumen eccentricity > 15%) were excluded from analysis. All dissections were performed by the same operator under standardized conditions to reduce procedural variability. Only specimens from animals with confirmed gestational age and without macroscopic vascular abnormalities were included. Samples showing mechanical damage during dissection or artifacts that could compromise histological evaluation were excluded prior to analysis. Immediately after dissection, aortic segments were gently rinsed in phosphate-buffered saline (PBS, pH 7.4; Gibco, Thermo Fisher Scientific, Waltham, MA, USA) to remove residual blood while preserving endothelial integrity. Tissues were then fixed by immersion in 5% neutral buffered formalin (pH 7.4) for 24 h at 4 °C, using a minimum fixative-to-tissue volume ratio of 10:1 to ensure optimal preservation of tissue architecture and extracellular matrix components [41]. Following fixation, samples were processed for paraffin embedding using an automated tissue processor (Leica TP1020, Leica Biosystems, Wetzlar, Germany). The protocol included dehydration through a graded ethanol series (70%, 80%, 90%, and 100%; 15 min each), clearing in xylene (three changes, 10 min each), and infiltration with molten paraffin (Paraplast Plus^®^, Sigma-Aldrich, St. Louis, MO, USA) at 60 °C for 4 h. Samples were consistently oriented to obtain comparable transverse sections of the vascular lumen, then embedded in paraffin blocks and allowed to solidify at room temperature. Serial sections of 5 µm thickness were obtained using a rotary microtome (Leica RM2235, Leica Biosystems). Initial sections were discarded until optimal morphological integrity was achieved. Sections were mounted on poly-L-lysine-coated glass slides (Sigma-Aldrich, St. Louis, MO, USA) to enhance adhesion during staining procedures. At least two non-consecutive sections were analyzed for each specimen, permitting independent evaluation and reducing potential bias associated with local tissue variability. All samples were processed under identical conditions and within the same batch to minimize technical variability associated with fixation, processing, and embedding procedures.

### 2.7. Histological Staining

For evaluation of the general morphology and elastic fiber organization of the aortic wall, transverse sections with a thickness of 5 μm were obtained from standardized anatomical segments and processed using conventional histological staining protocols. Sections were deparaffinized in xylene (three changes, 5 min each) and rehydrated through a descending ethanol series (100%, 95%, 80%, and 70%; 3 min each) to distilled water. For hematoxylin and eosin (H&E) staining, sections were incubated in Harris hematoxylin (Merck, Darmstadt, Germany) for 5 min, rinsed in running water, differentiated in 1% acid alcohol (10 s), and blued in Scott’s solution (pH 8.0) for 2 min. Subsequently, sections were counterstained with 1% alcoholic eosin (Sigma-Aldrich) for 2 min. In order to specifically visualize elastic fibers, serial sections were stained with 1% orcein in 70% ethanol (Sigma-Aldrich) for 60 min at 37 °C, followed by differentiation in 70% ethanol (1–2 min). This staining approach was selected due to its sensitivity for detecting elastic lamellae organization and fragmentation within the arterial wall. In both staining protocols, sections were dehydrated through an ascending ethanol series (70%, 95%, and 100%), cleared in xylene, and mounted using a synthetic resin medium (Entellan^®^; Merck KGaA, Darmstadt, Germany). All staining procedures were performed in parallel under standardized conditions of time, temperature, and reagent preparation. Sections from different experimental groups were processed simultaneously and randomly distributed across staining batches to minimize technical variability. Additionally, outcome assessment was conducted under blinded conditions. Internal controls, including sections from control animals, were used to verify staining consistency and quality according to established histological criteria [41].

### 2.8. Microscopic Evaluation and Image Analysis

Histological sections were examined using a Leica DM750 optical microscope (Leica Microsystems, Wetzlar, Germany) equipped with 10×, 20×, and 40× objectives (NA: 0.25, 0.40, and 0.65, respectively). Images were acquired using a Leica MC170 HD digital camera and Leica Application Suite software version 4.13 (Leica Microsystems, Wetzlar, Germany) under standardized conditions of illumination, white balance, and exposure. For each specimen, at least five non-overlapping microscopic fields per section were analyzed using a systematic random sampling approach along the vascular perimeter in order to ensure structural representativeness and reduce selection bias. All image acquisition and subsequent analyses, were performed blinded to experimental group allocation throughout, including qualitative histological assessment, morphometric measurements, and densitometric quantification. Group identity was concealed by coding slides with alphanumeric identifiers prior to analysis; codes were revealed only after all measurements had been recorded and exported.

### 2.9. Qualitative Evaluation

Qualitative assessment focused on the structural organization of the aortic wall, including the identification and integrity assessment of the tunica intima, media, and adventitia as well as the orientation and distribution of smooth muscle cells and extracellular matrix components. In H&E-stained sections, cellular density, presence of inflammatory infiltrate, continuity of vascular layers, and overall tissue preservation were evaluated. In orcein-stained sections, the organization, continuity, waviness, and spatial distribution of elastic lamellae within the tunica media were assessed along with their relative arrangement and structural integrity. Qualitative observations were conducted under blinded conditions and recorded as comparative morphological descriptions, emphasizing structural patterns and differences between experimental groups.

### 2.10. Quantitative Morphometric Analysis

Morphometric parameters included the thickness of the tunica intima, media, and adventitia as well as the structural density and spatial organization of elastic fibers. These parameters were quantified from standardized images using ImageJ software (version 1.53, NIH, Bethesda, MD, USA), previously calibrated with a micrometric scale (μm). For each specimen, multiple fields per section were analyzed and averaged to obtain a single representative value per animal. Variables reflecting luminal area or media-to-lumen ratio were not included in the morphometric analysis, as these parameters require distension-controlled or pressure-fixed tissue preparations in order to yield valid estimates of in vivo geometry [26]; the immersion fixation protocol employed in this study did not preserve physiological luminal dimensions, and their inclusion would have introduced a systematic bias unrelated to the experimental groups. The selected variables, tunica thicknesses, elastic fiber density and inter-fiber distance, were defined a priori as complementary descriptors of arterial wall structural integrity and spatial remodeling that were sufficient to address the primary morphometric aims of the study. Total wall thickness was defined as the distance between the luminal surface (intima) and the boundary of the adventitia. Measurements of tunica intima, media, and adventitia thickness were performed using clearly defined histological landmarks. At least three measurements per field were obtained and averaged. Structural density of elastic fibers was quantified by direct manual counting within defined regions of interest (ROI), including only orcein-positive fibers that exhibited morphological continuity and circumferential orientation. This approach was selected to ensure accurate identification of structurally preserved elastic lamellae. The area of each ROI was determined in ImageJ following pixel-to-micrometer calibration and fiber counts were normalized to a standard reference area of 10,000 μm^2^ to allow for cross-specimen comparability, yielding a density expressed as number of fibers per 10,000 μm^2^ (*n*/10,000 μm^2^).

All morphometric and densitometric measurements were performed under blinded conditions following a standardized protocol, consistent with the blinding procedure described in Section 2.5. To evaluate spatial organization of the elastic network, inter-fiber distance was measured. Consecutive pairs of elastic fibers within each ROI were identified and the Euclidean distance between their external boundaries was calculated. At least three measurements per field were obtained and averaged to generate a representative value per sample. This parameter was used as an indicator of structural remodeling of the elastic matrix. Morphometric variables were selected as complementary descriptors of arterial wall organization, allowing for assessment of both structural integrity (wall thickness and structural density of elastic fibers) and spatial remodeling (inter-fiber distance). To avoid pseudoreplication, all measurements obtained from multiple fields and sections were averaged at the individual level to yield a single representative value per animal for each analyzed parameter. The animal was considered the experimental unit for all statistical analyses.

### 2.11. Densitometric Analysis of Elastic Fibers

Densitometric analysis was performed to estimate the relative abundance of elastic fibers within the tunica media. Digital images were obtained using a 20× objective (NA 0.40) under constant acquisition settings. Images were processed in ImageJ following a standardized workflow. First, images were converted to 8-bit grayscale and background subtraction was applied to minimize staining variability. Five regions of interest (ROI; 100 × 100 μm) per section were selected using a systematic random approach within the tunica media, avoiding areas with artifacts, discontinuities, or vascular structures. Elastic fibers were segmented using the ImageJ “Default” automatic thresholding algorithm and visually validated for accurate segmentation of orcein-positive structures. Integrated optical density (IOD) was calculated as the sum of pixel intensity values within the segmented area and used as a proxy for elastic fiber content. All densitometric measurements were performed under blinded conditions using a consistent analysis protocol. Values obtained from multiple ROI were averaged to generate a single representative value per animal prior to statistical analysis.

### 2.12. Maternal Blood Pressure Measurement

Maternal systolic blood pressure (SBP), diastolic blood pressure (DBP), mean arterial pressure (MAP), and heart rate (HR) were assessed on gestational days 6, 12, and 18 post-fertilization (dpf) across all four experimental groups (FA/FA, FA/NFA, NFA/FA, and NFA/NFA) using a non-invasive tail-cuff plethysmography system (CODA^®^, Kent Scientific Corporation, Torrington, CT, USA) validated for use in rodents. Prior to data collection, animals underwent a three-day acclimation period consisting of daily 10-min sessions in restraint chambers in order to minimize stress associated with handling and the measurement procedure. During each measurement session, animals were positioned in cylindrical restraint holders under controlled environmental conditions (24 ± 1 °C; 12:12 h light/dark cycle). The tail was pre-warmed at 32 °C for 5 min using an integrated heating platform to improve signal detection, after which a pressure cuff and a volume pressure recording (VPR) sensor were placed at the base of the tail according to the manufacturer’s specifications. Each session consisted of ten consecutive measurement cycles; the first two were discarded to allow system stabilization, then the remaining eight were averaged to generate representative values for each hemodynamic parameter. Measurements with artifacts or excessive variability were automatically excluded based on system quality criteria. All sessions were conducted in a quiet environment within a standardized time window (09:00–11:00 h) to minimize circadian variability, and the system was calibrated prior to each session. As a quality control measure, repeated measurements in a randomly selected subset of animals (10% of the total sample) confirmed an intra-session variability of less than 5%. The operator responsible for data acquisition and analysis was blinded to experimental group allocation throughout the procedure.

### 2.13. Statistical Analysis

Aortic wall structural parameters were defined as the primary outcomes. The statistical analysis was structured into three hierarchical levels aligned with the primary, secondary, and exploratory aims. Primary outcomes comprised morphometric parameters of the aortic wall at gestational day 23 (tunica intima, media, and adventitia thickness; elastic fiber density; inter-elastic fiber distance). Data distribution was assessed using the D’Agostino–Pearson omnibus normality test. Densitometric parameters (integrated optical density in H&E- and orcein-stained sections) followed a normal distribution and were expressed as mean ± SD, then compared by one-way ANOVA with Holm–Šídák post hoc tests (FA/FA as reference); effect size was estimated using eta-squared ($\eta^{2}$) [42]. Morphometric variables violated normality assumptions; these variables were expressed as median (IQR; 25th–75th percentiles) and compared by Kruskal–Wallis test with Dunn’s post hoc adjustment, with effect size quantified using epsilon-squared ($\varepsilon^{2}$) derived from the *H* statistic. Ninety-five percent confidence intervals for group medians were estimated by bootstrapping (10,000 iterations). Although the design has an inherent 2 × 2 factorial structure (pregestational × gestational exposure), the four groups were treated as levels of a single categorical factor in order to maintain alignment with the primary objective of characterizing differences relative to a clean-air reference; the absence of a formal interaction term is acknowledged as an analytical limitation. Secondary outcomes (SBP, DBP, MAP, and HR at gestational days 6, 12, and 18 dpf) were summarized descriptively as mean ± SD to characterize temporal hemodynamic trajectories; no inferential comparisons were performed, as these variables were designated a priori as descriptive. Exploratory analyses included Spearman’s rank correlations ($r_{s}$) between morphometric variables (gestational day 23) and hemodynamic parameters (SBP, DBP, and HR at gestational day 18, selected as the closest time point to tissue collection; MAP was excluded to avoid redundancy) along with unsupervised principal component analysis (PCA). All variables were centered and scaled to unit variance; the first two components were retained (PC1: 28.8%; PC2: 20.4%; cumulative: 49.2%). These analyses were hypothesis-generating and not intended to support causal inference. All analyses were conducted at the individual-animal level. Measurements from multiple fields were averaged per animal to avoid pseudoreplication. Because PM_2.5_ exposure was applied at the chamber level, potential shared environmental variance was considered when interpreting group differences. All tests were two-tailed (α = 0.05). Morphometric and densitometric comparisons were performed using GraphPad Prism (version 10.0, GraphPad Software, San Diego, CA, USA). Hemodynamic data visualization, Spearman’s rank correlations, and PCA were conducted in *R* [43] (version 4.5.1), using the packages ggplot2 [44] for figure generation, FactoMineR [45] for PCA computation, and factoextra [46] for PCA visualization.

## 3. Results

### 3.1. Air Pollution Exposure

Second-generation (G2) female rats were continuously exposed to PM_2.5_ from conception through postnatal day 82. During the study period, the daily average ambient PM_2.5_ concentration was 48.8 μg/m^3^ (±36.1; 95% CI: 42–56), confirming environmentally relevant exposure levels consistent with biomass combustion settings (Supplementary Document S2). Within the exposure chambers, PM_2.5_ concentrations differed markedly: the NFA chamber averaged 44.6 μg/m^3^ (±9.8; 95% CI: 42.2–47.0), whereas the FA chamber was reduced to 3.0 μg/m^3^ (±1.3; p<0.001), representing a 94% attenuation. These conditions were consistently maintained throughout the experimental period, generating two clearly differentiated particulate matter environments. Temporal variation in PM_2.5_ concentrations is shown in Supplementary Document S3.

### 3.2. Histology

**Figure 2:**
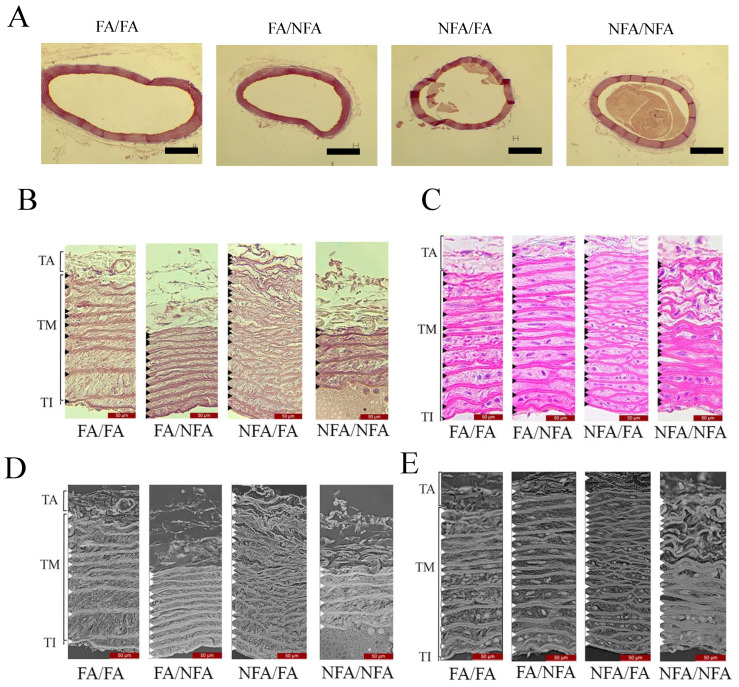
Representative sections of the aortic arch in pregnant rats from the experimental groups (FA/FA, FA/NFA, NFA/FA, and NFA/NFA). (**A**) Panoramic view of aortic arch cross-sections stained with orcein (scale bar = 300 μm). (**B**) Higher magnification of orcein-stained sections showing tunica adventitia (TA), tunica media (TM), and tunica intima (TI). (**C**) Higher magnification of hematoxylin–eosin (H&E)-stained sections showing the same vascular layers. In the FA/FA group (control), the tunica media exhibited regularly arranged parallel elastic lamellae. In the FA/NFA group, the lamellar organization was largely preserved, with mild variations in fiber compactness. In the NFA/FA group, regions with irregular organization of elastic lamellae and the presence of amorphous extracellular material were observed. In the NFA/NFA group, more extensive changes in lamellar architecture were evident, characterized by increased waviness and disorganization of elastic fibers. (**D**,**E**) Densitometric analysis of the aortic wall in the same experimental groups, based on sections stained with orcein (**D**) and H&E (**E**). The integrated optical density (IOD) of the tunica media was quantified using digital image analysis (ImageJ, version 1.53), with n=12 per group. Statistical analysis was performed as described in Table 1. Experimental groups were defined as follows: FA/FA (filtered air during development and gestation), FA/NFA (filtered air during development and non-filtered air during gestation), NFA/FA (non-filtered air during development and filtered air during gestation), and NFA/NFA (non-filtered air during both periods). Scale bar for panels (**B**–**E**) = 50 μm. Abbreviations: FA, filtered air; NFA, non-filtered air.

Histological evaluation of the aortic arch in pregnant rats exposed to different filtered (FA) and non-filtered air (NFA) regimens during pregestational and gestational stages revealed group-dependent morphological variations. In the FA/FA group (control), the aortic wall architecture was preserved. The tunica intima consisted of a continuous endothelial monolayer with basophilic nuclei and no evident structural changes. The tunica media displayed regularly arranged concentric fenestrated elastic lamellae, as evidenced by orcein staining, interspersed with smooth muscle cells and without signs of fragmentation. The tunica adventitia was composed of loosely organized connective tissue with irregular collagen fibers and scattered vasa vasorum, without cellular infiltrates. In the FA/NFA group exposed to non-filtered air during gestation, the tunica media maintained its concentric organization of elastic lamellae and smooth muscle cells; however, regional variations in the density and spacing of elastic lamellae were observed. The tunica adventitia exhibited areas of reduced collagen organization with heterogeneous staining patterns, without evidence of inflammatory cell accumulation. In the NFA/FA group that was exposed during the pregestational stage, structural alterations were observed in the tunica media, including focal fragmentation of elastic lamellae and the presence of amorphous extracellular material, while the tunica intima remained structurally continuous. The tunica adventitia showed irregular arrangement of connective tissue fibers and variable staining intensity. In the NFA/NFA group, which was subjected to continuous exposure, more pronounced structural heterogeneity was observed. The tunica media exhibited regions with variable compactness and discontinuities in elastic lamellae, along with disorganization of extracellular matrix components, while the tunica adventitia presented irregular collagen organization and heterogeneous staining patterns (Figure 2).

**Table 1:** Integrated optical density (IOD) of the tunica media of the aortic arch in pregnant rats.IOD was assessed by digital densitometric analysis in sections stained with hematoxylin–eosin (H&E) and orcein. Values are expressed as mean ± standard deviation (n=12 per group). Group comparisons were performed using one-way ANOVA followed by Holm–Šídák post hoc testing, using FA/FA as the reference group. No statistically significant differences were observed among groups (p>0.05).

Staining	FA/FA	FA/NFA	NFA/FA	NFA/NFA	ANOVA *p*	Holm–Šídák (vs. FA/FA)
H&E	49.05±5.07	47.96±5.46	50.27±6.16	54.24±3.64	0.1040	ns
Orcein	88.13±4.44	90.54±7.89	85.93±4.30	90.52±7.09	0.4057	ns

Abbreviations: FA, filtered air; NFA, non-filtered air; IOD, integrated optical density; H&E, hematoxylin–eosin; ns, not significant.

### 3.3. Densitometric Analysis

Densitometric quantification of the tunica media by integrated optical density (IOD) revealed no statistically significant differences among groups in either H&E- (p=0.1040) or orcein-stained sections (p=0.4057; Table 1). Effect sizes were negligible for both staining protocols ($\eta^{2}<0.06$), indicating that PM_2.5_ exposure produced no detectable change in the overall optical density of the tunica media under the analyzed conditions, regardless of timing or duration (Figure 2).

### 3.4. Aortic Arch Morphometry

**Figure 3:**
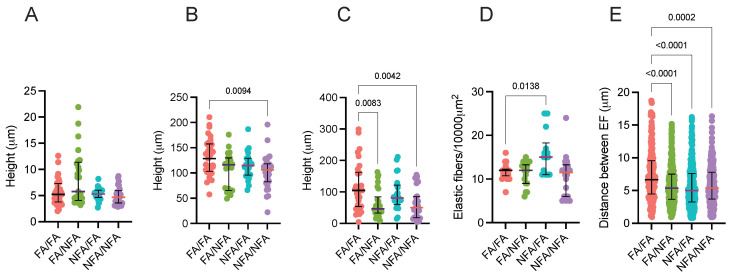
Morphometric analysis of the maternal aortic arch in pregnant rats exposed to PM_2.5_. Scatter plots showing individual values and distributions of morphometric parameters in four experimental groups (n=12 per group): FA/FA (filtered air during development and gestation), FA/NFA (filtered air during development and non-filtered air during gestation), NFA/FA (non-filtered air during development and filtered air during gestation), and NFA/NFA (non-filtered air during both periods). (**A**) Thickness of the tunica media (μm). (**B**) Thickness of the tunica intima (μm). (**C**) Thickness of the tunica adventitia (μm). (**D**) Elastic fiber density, expressed as the number of fibers per 10,000 µm^2^ of tunica media (orcein staining). (**E**) Distance between elastic fibers (EF, μm), defined as the mean spacing between consecutive elastic fibers within the tunica media. Measurements were obtained from histological sections stained with hematoxylin–eosin (H&E) and orcein, and were quantified using ImageJ (version 1.53). Statistical significance was assessed using the Kruskal–Wallis test followed by Dunn’s multiple comparisons test, with FA/FA as the reference group. Exact *p*-values are indicated in the figure; statistical significance was set at p<0.05.

Morphometric analysis of the aortic arch, which forms the primary outcome of this study, revealed group-dependent structural differences in four of five assessed parameters (Figure 3; Table 2). The complete dataset is provided in Supplementary Document S4. Tunica intima thickness did not differ significantly among groups (KW: p=0.0995; $\varepsilon^{2}=0.07$). Tunica media thickness differed significantly (KW: p=0.0251; $\varepsilon^{2}=0.09$), with the FA/FA group exhibiting the highest median (128 μm; IQR: 103–158) and the NFA/NFA group the lowest (106 μm; IQR: 83–118). A significant pairwise difference was confirmed by post hoc analysis (p=0.0094); comparisons with FA/NFA and NFA/FA did not reach significance. Tunica adventitia thickness also differed significantly (KW: p=0.0014; $\varepsilon^{2}=0.14$). The FA/FA group showed the highest median (105 μm; IQR: 54–163), whereas FA/NFA and NFA/NFA exhibited the greatest reductions (medians: 46 and 50 μm, respectively; post hoc: p=0.0083 and p=0.0042). NFA/FA presented an intermediate value that did not differ significantly from FA/FA. Elastic fiber density differed significantly across groups (KW: p=0.0038; $\varepsilon^{2}=0.13$), with NFA/FA showing the highest median density (15 fibers/10,000 μm^2^; IQR: 11–18) relative to FA/FA (12; IQR: 11–12); post hoc analysis confirmed a significant difference only between these two groups (p=0.0138). Inter-elastic fiber distance showed the most pronounced group differences (KW: p<0.0001; $\varepsilon^{2}=0.19$); the FA/FA group exhibited the highest median spacing (6.7 μm; IQR: 4.5–9.6), whereas all PM_2.5_-exposed groups showed consistently lower values irrespective of exposure timing. All pairwise comparisons with FA/FA were statistically significant (p<0.0001 for FA/NFA and NFA/FA; p=0.0002 for NFA/NFA).

### 3.5. Maternal Blood Pressure and Heart Rate

**Figure 4:**
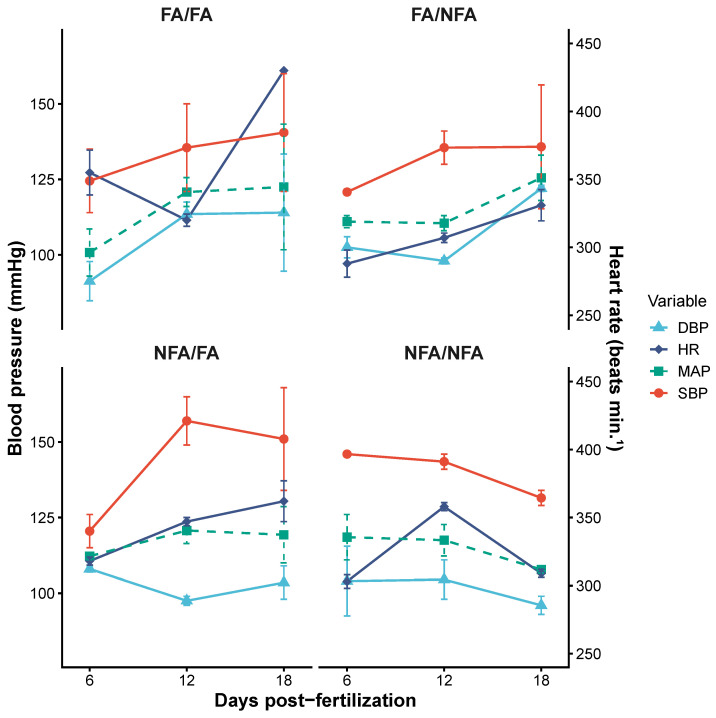
Maternal blood pressure and heart rate in pregnant rats exposed to PM_2.5_ at different gestational time points. Systolic blood pressure (SBP, mmHg), diastolic blood pressure (DBP, mmHg), mean arterial pressure (MAP, mmHg), and heart rate (HR, beats per minute) were measured at gestational days 6, 12, and 18 post-fertilization (dpf) in four experimental groups (n=12 per group): FA/FA (filtered air during development and gestation), FA/NFA (filtered air during development and non-filtered air during gestation), NFA/FA (non-filtered air during development and filtered air during gestation), and NFA/NFA (non-filtered air during both periods). Measurements were obtained using a non-invasive CODA tail-cuff system (Kent Scientific) in which a pressure cuff was placed on the tail to detect blood flow in the caudal artery. Data are presented as mean ± standard deviation. Abbreviations: FA, filtered air; NFA, non-filtered air; dpf, days post-fertilization; SBP, systolic blood pressure; DBP, diastolic blood pressure; MAP, mean arterial pressure; HR, heart rate.

Hemodynamic parameters (SBP, DBP, MAP, and HR) constituted secondary descriptive outcomes and were not subjected to inferential comparison. Temporal trajectories across gestational days 6, 12, and 18 post-fertilization are shown in Figure 4; individual group values are provided in Supplementary Document S5. In the FA/FA group, SBP increased progressively from day 6 to day 18 (120.8 to 135.8 mmHg); HR showed a non-monotonic pattern with a transient decrease at day 12 (307 bpm), followed by a marked rise at day 18 (331 bpm), consistent with physiological cardiovascular adaptation during normal pregnancy. In the FA/NFA group, SBP peaked at day 12 (157.0 mmHg) and remained elevated at day 18 (151.0 mmHg), with both values exceeding 140 mmHg; HR increased progressively throughout gestation (318, 347, and 362 bpm). In the NFA/FA group, SBP increased sharply between days 6 and 12 (124.5 to 157.0 mmHg), with partial attenuation at day 18 (151.0 mmHg); HR reached the highest value recorded across all groups at day 18 (430 bpm). In the NFA/NFA group, the highest SBP was observed at day 6 (146.0 mmHg) and declined progressively through days 12 and 18 (143.5 and 131.5 mmHg, respectively), while HR showed a transient rise at day 12 (358 bpm) followed by a return to near-baseline values (309 bpm). Each PM_2.5_-exposed group exhibited a qualitatively distinct temporal hemodynamic trajectory relative to FA/FA, with differences in both magnitude and direction of change across gestation, suggesting a pattern of maternal hemodynamic variation dependent on the exposure window. As an integrated index of myocardial oxygen demand, we calculated the rate-pressure product (RPP = HR × SBP/1000) for all time points and exposure groups (Supplementary Document S6). RPP was significantly elevated in FA/NFA and NFA/NFA groups at GD12 (FA/NFA: 54.46 ± 3.21; NFA/NFA: 51.25 ± 2.47) compared to FA/FA controls (41.54 ± 2.15; one-way ANOVA p<0.001; Holm-Šídák post hoc p<0.01 for both comparisons). Similarly, elevated RPP persisted in FA/NFA and NFA/FA groups at GD18, consistent with increased cardiovascular workload during mid-to-late gestation in animals exposed to unfiltered air during either exposure window.

### 3.6. Spearman Correlation Analysis

Associations between significant structure and function were identified in two experimental groups (Table 2); all pairwise correlations are provided in Supplementary Document S7. In the FA/FA group, tunica adventitia thickness showed a moderate positive correlation with SBP ($r_{s}=0.6014$; p=0.0428). In the FA/NFA group, two opposing associations with SBP were identified: tunica media thickness was positively correlated ($r_{s}=0.7063$; p=0.0129), whereas tunica adventitia thickness was negatively correlated ($r_{s}=-0.7273$; p=0.0096). No significant associations with DBP or HR were identified in any group, and no significant correlations were observed in the NFA/FA or NFA/NFA groups.

### 3.7. Multivariate Structure of Aortic and Hemodynamic Variables

**Figure 5:**
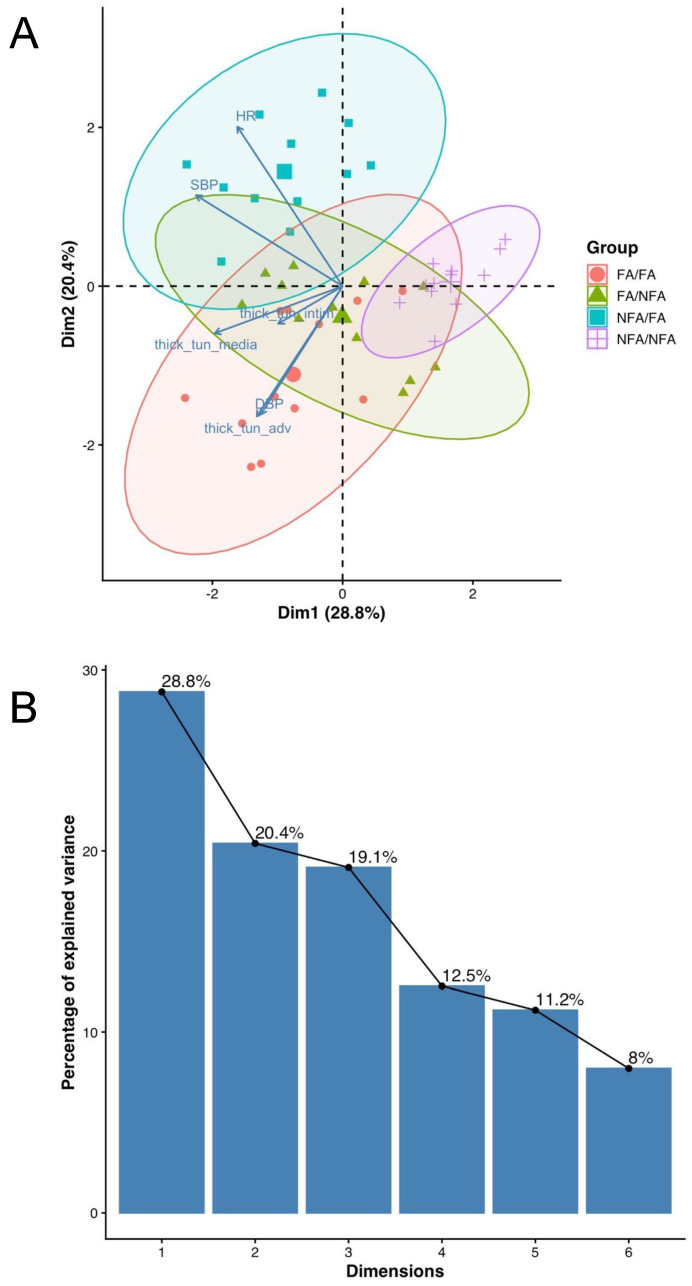
Principal component analysis of aortic structural and hemodynamic variables. (**A**) PCA biplot illustrating the multivariate distribution of individual samples according to experimental groups (FA/FA, FA/NFA, NFA/FA, and NFA/NFA). Each point represents one individual, and colored ellipses indicate the 95% confidence regions for each group. Arrows represent variable loadings, showing the direction and magnitude of the contribution of aortic structural parameters (tunica intima, tunica media, and tunica adventitia thickness) and hemodynamic variables (systolic blood pressure, diastolic blood pressure, and heart rate) to the principal components. Principal components 1 (PC1) and 2 (PC2) explain 28.8% and 20.4% of the total variance, respectively. (**B**) Scree plot showing the percentage of variance explained by each principal component. The first two components accounted for 49.2% of the total variance, supporting their use for dimensionality reduction and visualization of multivariate patterns.

Principal component analysis (PCA) was performed as an exploratory visualization of multivariate relationships among morphometric and hemodynamic variables (Figure 5). The first two principal components accounted for 49.2% of total variance (PC1: 28.8%; PC2: 20.4%). Biplot visualization revealed partial separation of exposure groups in the PC1–PC2 plane, with FA/FA animals clustering predominantly toward the negative axis of PC1 and NFA/FA animals showing displacement toward the positive axis of PC2. However, substantial overlap was observed among confidence ellipses for the FA/NFA, NFA/FA, and NFA/NFA groups, indicating considerable within-group heterogeneity. Variable loadings on PC1 were dominated by tunica media thickness, adventitia thickness, DBP and SBP (negative loadings), and tunica intima thickness (positive loading), suggesting that PC1 primarily captures a gradient from vessel wall thinning to hemodynamic stress. PC2 loadings were driven by heart rate (positive loading) and elastic fiber density. These patterns suggest exposure window-dependent structural and hemodynamic profiles; however, the modest variance explained by the first two components (49.2%) and the substantial overlap among confidence ellipses indicate that PCA should be interpreted as an exploratory pattern visualization rather than as definitive evidence of discrete group stratification.

## 4. Discussion

### 4.1. Exposure and Validation of the Experimental Model

PM_2.5_ concentrations in the non-filtered air (NFA) chamber averaged 44.6 μg/m^3^ (CV = 21.9%). These values are environmentally representative of the winter context of Temuco, where daily concentrations may exceed 150 μg/m^3^ [6,7] and consistently surpass WHO daily and annual limits (15 and 5 μg/m^3^, respectively [1]). The filtration system reduced PM_2.5_ in the FA chamber to 3.0 μg/m^3^, an approximate 94% reduction, consistent with references obtained using high-efficiency HEPA filtration [47,48]. This indicates that a robust exposure contrast was generated, supporting interpretation of the observed biological differences in relation to PM_2.5_ as the primary differential exposure component between chambers. A central strength of the model is that exposure occurs through inhalation of unmodified ambient air, preserving the real physicochemical composition of PM_2.5_ derived from wood smoke without artificial concentration or intravascular instillation [23,24,36]. This design replicates the human exposure route, allowing for the evaluation of chronic systemic effects under ecologically valid conditions [49]. Because the filtration system selectively removes particulate matter without affecting the gaseous fraction, as reflected in the absence of differences in CO and NO_2_ between chambers, particulate matter represents the main differential exposure component, although contributions from the gaseous fraction common to both chambers cannot be fully excluded [50,51]. The multigenerational design in which G2 rats accumulated exposure across G0 and G1 lineages from conception onward constitutes a validated model of chronic exposure that captures cumulative effects across critical developmental windows [35,52]. Although mechanistic pathways were not directly assessed, the observed structural and hemodynamic patterns support the need for future studies addressing vascular signaling and oxidative stress pathways.

### 4.2. Structural Remodeling of the Aortic Arch

#### 4.2.1. Histological Changes

Histological evaluation of the aortic arch revealed a gradient of morphological changes dependent on the window of PM_2.5_ exposure. The non-exposed group (FA/FA) exhibited preserved aortic architecture, with regularly arranged concentric elastic lamellae in the tunica media and a normally composed adventitia, validating the structural integrity of the experimental model. The group exposed exclusively during gestation (FA/NFA) showed regional variations in the density and spacing of elastic lamellae along with focal disorganization of adventitial collagen and without inflammatory infiltrates. These findings indicate that restricted gestational exposure is sufficient to modify extracellular matrix organization, consistent with evidence identifying gestation as a critical window of vascular susceptibility [15,16]. The group that was exposed exclusively during the pregestational stage (NFA/FA) exhibited a particularly distinctive structural pattern, characterized by focal fragmentation of elastic lamellae and the presence of amorphous extracellular material despite the absence of active gestational exposure. This finding is consistent with the possibility that pregestational exposure may be associated with a persistent structural imprint that is not reversed during gestation under filtered air conditions, consistent with the concept of prenatal vascular programming [35,53]. The group subjected to continuous pregestational and gestational exposure (NFA/NFA) presented the greatest structural heterogeneity, with discontinuities in elastic lamellae and extracellular matrix disorganization affecting both tunicae, suggesting an additive effect of sustained exposure. From a biological plausibility perspective, the observed elastic fragmentation is consistent with previously described pathways involving consistent with pathways involving extracellular matrix remodeling previously described for PM_2.5_ exposure under PM_2.5_-induced oxidative stress, which may generate elastokines associated with vascular smooth muscle cell apoptosis [20,54]. However, as these processes were not directly assessed in the present study, this interpretation should be considered a biologically plausible inference rather than direct mechanistic evidence, potentially contributing to reduced adaptive capacity of the tunica media during gestation [16].

#### 4.2.2. Densitometry

Densitometric quantification of the tunica media did not reveal significant differences among groups in either H&E-stained (p=0.1040) or orcein-stained sections (p=0.4057). Rather than reflecting a methodological limitation, this finding is structurally informative, indicating that PM_2.5_ exposure did not reduce total elastin content but instead altered its spatial organization, architectural continuity, and lamellar distribution. This distinction is functionally relevant, as aortic distensibility depends not only on elastin quantity but also on its concentric lamellar organization and three-dimensional integrity [26,54]. The histologically-observed focal fragmentation and reduced inter-fiber spacing are sufficient to alter wall biomechanics even in the absence of net elastic mass loss [26,53].

#### 4.2.3. Morphometry

Morphometric analysis identified significant group differences in tunica media (KW: p=0.0251) and tunica adventitia thickness (KW: p=0.0014), with progressively lower values in the exposed groups relative to FA/FA; no significant differences were observed in the tunica intima (p=0.0995). Medial thinning may reflect disruption of the adaptive thickening that normally occurs in the pregnant aorta (reported at ∼12–14% in murine models [16]), potentially associated with vascular smooth muscle cell alterations previously described under PM_2.5_ exposure [19,20]. Adventitial thinning, significant in gestational (FA/NFA; p=0.0083) and continuous exposure groups (NFA/NFA; p=0.0042) but not in the pregestational-only group (NFA/FA), is consistent with greater adventitial susceptibility during active gestational exposure, potentially related to fibroblast alterations associated with pro-inflammatory and oxidative stimuli [54,55,56]. The absence of intimal differences is consistent with the vascular biology of PM_2.5_, the endothelial effects of which predominantly manifest as functional alterations, reduced nitric oxide bioavailability, and NF-κB activation rather than morphometrically detectable structural changes [3,4,19]; any underlying endothelial dysfunction was not directly assessed in the present study. In this context, the distinction between load-dependent and intrinsic structural stiffness is relevant; evidence from women with a history of preeclampsia demonstrates that elevated aortic stiffness is attributable predominantly to the load-dependent component and associates with reduced cardiovagal baroreflex sensitivity [57]. The parietal thinning documented here may constitute a morphological substrate that reduces passive compliance, thereby transferring hemodynamic regulation towards the load-dependent component, although this hypothesis requires direct evaluation in future studies. Taken together, these findings are consistent with a pattern of aortic remodeling characterized by elastic disorganization without significant mass loss and exposure window-dependent thinning of the tunica media and adventitia.

An important interpretive consideration concerns the potential contribution of PM_2.5_-associated endocrine disruption to the structural differences described above. Ovarian hormone concentrations (progesterone and estradiol) and reproductive outcomes in G2 females from the same experimental model have been previously reported [24]. That study found no significant differences in circulating hormone levels among experimental groups, indicating that hypothalamic–pituitary–ovarian axis function was not substantively altered under the exposure conditions they evaluated. This observation is consistent with the interpretation that the aortic structural differences documented in the present study are unlikely to be secondary to PM_2.5_-mediated hormonal disruption, and supports their association with non-endocrine pathways such as oxidative stress and extracellular matrix remodeling [20,54]. Nevertheless, we cannot fully exclude subtle hormonal fluctuations below the detection threshold of the employed assays or effects mediated by local paracrine signaling not captured by circulating concentrations.

### 4.3. Maternal Hemodynamic Response: Blood Pressure and Heart Rate During Gestation

All four experimental groups exhibited qualitatively distinct hemodynamic trajectories throughout gestation, reflecting differences in both the timing and duration of PM_2.5_ exposure. The non-exposed group (FA/FA) displayed the expected reference pattern of progressive moderate increase in SBP from 120.8 ± 1.0 mmHg at day 6 to 135.8 ± 20.5 mmHg at day 18, with parallel HR increase from 288.0 ± 10.0 bpm at day 6 to 331.0 ± 11.5 bpm at day 18, which is consistent with normal gestational cardiovascular physiology in normotensive Sprague–Dawley rats [16,58,59].

The group exposed exclusively during gestation (FA/NFA) exhibited the most pronounced SBP increase, reaching 151.0 ± 17.0 mmHg at day 18, exceeding the diagnostic threshold for gestational hypertension (140 mmHg; [58,60]) and with concomitant HR elevation to 362.0 ± 15.0 bpm at day 18, compatible with a pattern that may reflect increased sympathetic activity [61]. This profile aligns with mechanisms described in PM_2.5_-associated gestational hypertension, namely, ROS-mediated reduction of endothelial nitric oxide bioavailability, hypothalamic activation increasing sympathetic tone, and systemic inflammatory responses elevating peripheral vascular resistance [3,4,14,61,62], although these were not directly assessed in the present study.

The group exposed exclusively during the pregestational stage (NFA/FA) exhibited the most distinct structural pattern: SBP already elevated at day 6 (124.5 ± 10.5 mmHg) despite gestation under low-exposure conditions and the highest HR recorded at day 18 (430.0 ± 5.0 bpm), suggesting persistent hemodynamic programming, including potential sympathetic overactivation, that was maintained in the absence of active exposure. This interpretation is consistent with pathways previously described in response to PM_2.5_ exposure following preconceptional PM_2.5_ exposure [35,63] as well as with epidemiological evidence identifying the periconceptional period as an independent susceptibility window [12]. Notably, prepregnancy aortic stiffness, measured as aortic-popliteal pulse wave velocity, is elevated before clinical manifestation of pre-term preeclampsia, suggesting that vascular structural changes established prior to conception can independently predict gestational hemodynamic complications [64]. The hemodynamic trajectory of NFA/FA is consistent with this concept, supporting the hypothesis that pregestational PM_2.5_ exposure may be associated with the presence of a structural imprint that could influence cardiovascular adaptations which condition cardiovascular adaptation during subsequent pregnancy.

The group subjected to continuous exposure (NFA/NFA) presented the most divergent profile: SBP already exceeding the gestational hypertension threshold at day 6 (146.0 ± 0.5 mmHg; [58]), followed by a tendency toward normalization at day 18 (131.5 ± 2.5 mmHg). This paradoxical trajectory may reflect compensatory gestational adaptations combined with increased passive distensibility of the morphometrically thinned aortic wall documented in this group, potentially reducing late gestational SBP at the expense of diminished pulse buffering capacity. This is analogous to mechanisms described in models of aortic medial atrophy [16,20,26,65,66]. The concomitant MAP reduction (118.5 to 107.8 mmHg) and modest HR variation suggest that late SBP normalization reflects complex hemodynamic dysregulation rather than complete adaptive recovery, with functional implications that cannot be fully determined from the variables assessed in this study. These patterns are further supported by evidence that pulse wave velocity and augmentation index are significantly elevated in pregnancies complicated by preeclampsia relative to normotensive controls [67], positioning the structural aortic changes documented here as plausible morphological substrates of the hemodynamic alterations observed across exposure groups.

### 4.4. Pregestational Programming and Persistent Vascular Remodeling

A particularly noteworthy finding is the persistence of aortic structural alterations and hemodynamic changes in the NFA/FA group, despite transfer of this group to filtered air at the onset of gestation. This group exhibited reduced tunica media thickness, increased elastic fiber density, decreased inter-elastic fiber distance, and elevated heart rate at GD18—a phenotype resembling that of the continuously exposed NFA/NFA group—even after 23 days of filtered air exposure during gestation. This observation raises the possibility of irreversible vascular programming induced during the pregestational exposure window, and has important mechanistic and translational implications. Three potential mechanisms may underlie this structural and functional persistence. First, pregestational PM_2.5_ exposure may induce stable epigenetic modifications in vascular smooth muscle cells or endothelial cells. Prenatal environmental exposure alters DNA methylation patterns in cardiovascular tissues, creating heritable transcriptional states that persist beyond the exposure period [68,69]. Such epigenetic marks can affect genes regulating extracellular matrix turnover, vascular tone, and inflammatory signaling, establishing a pro-remodeling phenotype that is resistant to environmental improvement. Second, extracellular matrix reorganization established during pregestational exposure may create mechanical constraints that limit subsequent adaptive capacity. Maternal and neonatal exposure to environmental pollutants targets pro-inflammatory genes in arterial tissue and triggers early vascular remodeling that predisposes to subsequent disease [70]. When elastic fibers become fragmented and collagen architecture is disrupted, restoration of the native structure requires prolonged tissue turnover exceeding the gestational window available for recovery in our model. Third, pregestational exposure may alter autonomic nervous system development or vascular sympathetic innervation patterns. Developmental environmental exposures can program persistent autonomic dysfunction, including sustained sympathetic overactivity and altered cardiovascular reflex control that persist into adulthood [71]. The elevated heart rate observed in NFA/FA animals at GD18 is consistent with the programmed sympathetic drive established during the pregestational period. These proposed mechanisms are not mutually exclusive and likely operate synergistically. Critically, this finding has profound environmental health implications; if pregestational PM_2.5_ exposure establishes vascular alterations resistant to short-term environmental improvement, then public health interventions focused solely on reducing air pollution exposure *during* pregnancy may be insufficient to prevent adverse maternal cardiovascular outcomes. Effective prevention strategies may require sustained air quality improvement during women’s reproductive years *prior to conception* in order to avoid irreversible vascular programming. This underscores the importance of long-term environmental policy aimed at protecting cardiovascular health across the reproductive lifespan, consistent with the Developmental Origins of Health and Disease (DOHaD) framework [72].

### 4.5. Structure–Function Integration: Spearman Correlations and PCA as Exploratory Evidence of Exposure-Dependent Vascular–Hemodynamic Patterns

Spearman correlation analysis revealed that associations between aortic wall thickness and SBP varied qualitatively according to exposure history. In the non-exposed group (FA/FA), a moderate positive correlation between adventitial thickness and SBP ($r_{s}=0.6014$, p=0.0428) is consistent with the physiological relationship expected in normotensive pregnancy, where mechanical pressure stimuli are associated with extracellular matrix turnover and maintenance of wall tension [73]. In contrast, FA/NFA exhibited a divergent pattern in which tunica media thickness was positively correlated with SBP ($r_{s}=0.7063$, p=0.0129), while adventitial thickness showed a negative correlation ($r_{s}=-0.7273$, p=0.0096). The coexistence of opposite-sign correlations within the same group suggests a potential dissociation between medial and adventitial structural responses associated with gestational PM_2.5_ exposure, consistent with vascular remodeling under inflammatory and oxidative conditions [19,74]; however, this interpretation should be treated cautiously given the observational nature of the analysis. No significant correlations were identified in the NFA/FA or NFA/NFA groups, possibly reflecting increased intragroup variability consistent with the histological heterogeneity documented in these groups and sample size limitations. The temporal mismatch between morphometric (day 23) and hemodynamic (day 18) data constitutes an inherent design constraint that should be considered when interpreting these associations.

PCA provided a complementary multivariate exploratory description of the data structure [75]. The first two principal components explained 49.2% of total variance (PC1: 28.8%; PC2: 20.4%), leaving more than half of the variance unexplained and suggesting that additional latent factors not captured by the measured variables may contribute to group differences. Biplot visualization suggested partial separation along PC1, with groups having gestational NFA exposure (FA/NFA and NFA/NFA) tending towards positive scores and those with gestational FA exposure (FA/FA and NFA/FA) tending towards negative scores. However, substantial overlap of confidence ellipses was observed, particularly among the FA/NFA, NFA/FA, and NFA/NFA groups. Along PC2, the NFA/FA group was distributed toward positive scores, reflecting its distinct pregestational exposure profile, whereas the remaining groups were clustered near or below the PC2 axis. Tunica media and adventitia thickness, SBP, and DBP all contributed predominantly along the negative axis of PC1, while HR contributed primarily along the positive axis of PC2 and tunica intima thickness showed a loading along the positive axis of PC1 in a direction opposite to the remaining structural variables. These loading patterns suggest coordinated co-variation between aortic wall layers and hemodynamic variables; however, the modest variance explained by the first two components and the substantial overlap among groups indicate that PCA should be interpreted as an exploratory visualization of multivariate patterns rather than as confirmatory evidence of exposure window-dependent group stratification. PCA does not establish causal or mechanistic relationships between parameters, and the observed patterns may reflect common upstream factors (e.g., systemic inflammation, oxidative stress) rather than direct structure–function linkages. Future studies incorporating direct measurements of vascular stiffness, endothelial function, and molecular markers of remodeling will be necessary to clarify the biological basis of these associations [47,76,77,78].

### 4.6. Biomedical Relevance of the Findings

The findings of the present study suggest that the aortic arch may represent a potential target of exposure to PM_2.5_ derived from biomass combustion, which would extend the current experimental understanding of air pollution effects on gestational health beyond placental and fetal compartments. This observation is particularly relevant in southern Chilean cities such as Temuco, Osorno, and Coyhaique, where residential wood combustion leads to PM_2.5_ concentrations that consistently exceed World Health Organization guidelines during winter and where a substantial proportion of the population corresponds to women of reproductive age [6,7]. Within this environmental context, the structural alterations and hemodynamic patterns described in this experimental model are biologically plausible, although not directly generalizable to human populations. The observation that the pregestational period may represent a window of susceptibility, with structural and hemodynamic differences detectable even in animals gestating under filtered conditions (NFA/FA group), raises relevant considerations from an environmental health perspective. However, these findings should be interpreted cautiously, as they derive from a controlled experimental model and do not establish persistence or causality of effects across exposure windows. Nevertheless, they support the need to consider preconceptional exposure in populations chronically exposed to biomass-derived PM_2.5_, as interventions focused exclusively on gestational exposure may not fully capture the temporal complexity of environmental risk [12,35,52]. The multivariate patterns identified in this study suggest that structural and hemodynamic variables co-vary in an exposure-dependent manner. In this context, non-invasive indices of arterial stiffness, such as carotid–femoral pulse wave velocity and augmentation index, could represent potential functional correlates of vascular alterations associated with environmental exposure, as these measures reflect biomechanical properties of the arterial wall similar to those assessed morphometrically [22,77]. However, their validity as clinical biomarkers in the context of biomass-derived PM_2.5_ exposure during pregnancy remains to be established in translational human studies. In the context of climate change, with projected increases in biomass combustion episodes in multiple regions worldwide [3,4], these findings provide experimental evidence that may inform hypothesis generation regarding maternal cardiovascular vulnerability under chronic environmental exposure. The present study underscores the importance of incorporating maternal vascular health into environmental health research frameworks while also highlighting the need for future studies integrating clinical, physiological, and molecular approaches to validate and extend these observations in human populations.

### 4.7. Study Limitations

First, extrapolation to human physiology requires caution in light of differences between species in aortic wall proportions, elastic lamellar density, and hormonal regulation [26], although partial conservation of molecular pathways (oxidative stress, matrix metalloproteinase activation, VSMC apoptosis, RAAS modulation) suggests potential translational relevance [19,20]. Blood pressure assessment at only three gestational time points limits temporal resolution, precluding longitudinal modeling; these data were interpreted descriptively. Morphological measurements at GD22 occurred five days after peak blood pressure at GD18. While vascular remodeling lags hemodynamic perturbations due to slow matrix turnover [16,74,79,80] and persists beyond acute hemodynamic normalization [79], this offset may affect correlations. Blood pressure remained elevated at GD22, suggesting sustained stress, and aortic remodeling is unlikely to change substantially within this late-gestational interval [81], consistent with cross-sectional correlation approaches [82]. Second, tail-cuff plethysmography is sensitive to handling stress and lacks continuous circadian capture [83,84,85]. While three-day acclimatization, standardized protocols, and initial cycle exclusion yielded intra-session variability below 5%, residual bias cannot be excluded. Chamber-level exposure limits statistical independence among animals. No formal a priori sample size calculation was performed; although our approach was sufficient to detect significant structural differences (tunica media: p=0.0251; adventitia: p=0.0014), future power-based estimation is warranted. Third, source-specific organic markers (levoglucosan, methoxyphenols, polycyclic aromatic hydrocarbons) were not measured. Prior characterization of Las Encinas identified residential wood burning as the predominant winter source, and elemental analysis aligned with biomass combustion [24]; however, absence of concurrent organic profiling limits definitive attribution. The estrous cycle was not monitored; natural variability may have contributed to hemodynamic variance during early gestation. Ambient CO levels (0.4–0.8 ppm) were below Chilean standards (9 ppm) and WHO guidelines (10 ppm) and unlikely to produce independent effects, though interactive effects with trace co-pollutants (nitrogen oxides, volatile organic compounds) contributing synergistically through oxidative or inflammatory pathways cannot be excluded. Fourth, elastic fiber fragmentation and waviness lacked systematic quantitative scoring; although we report density and inter-fiber distance, fragmentation indices would quantify lamellar degradation more directly. Verhoeff–Van Gieson staining was not employed; while orcein provides superior specificity and photomicrographic clarity, VVG would enable simultaneous elastic fiber–collagen visualization for integrated matrix assessment. This study focused exclusively on the aortic arch; thus, regional differences in hemodynamic stress, elastin-to-collagen ratios, and smooth muscle phenotypes limit its generalization to other vascular territories. Fifth, hemodynamic assessment was limited to discrete blood pressure and heart rate measurements at three time points without pre-pregnancy baselines or continuous monitoring. Comprehensive maternal cardiovascular characterization would require cardiac performance indices (stroke volume, cardiac output), vascular parameters (total peripheral resistance, pulse pressure), and temporal dynamics that were not captured in this study [86,87]. Continuous monitoring can provide superior pattern characterization, capturing circadian variations and transient episodes [88,89]. Stroke volume, cardiac output, and total peripheral resistance, which are essential for differentiating resistance-driven versus flow-driven alterations [90], were not measured; this limits mechanistic interpretation of whether changes resulted from cardiac performance, vascular tone, or both [91]. Future studies incorporating continuous monitoring, echocardiography, and arterial tonometry are necessary to characterize the mechanisms underlying PM_2.5_-induced vascular remodeling. Sixth, functional vascular assessments were not performed. Comprehensive vascular characterization requires integration of structural parameters with functional measures of stiffness, vasoreactivity, and endothelial function [92,93]. Arterial stiffness (pulse wave velocity, distensibility) provides critical mechanical property information that is not inferable from morphometry and is independently associated with cardiovascular risk [92,93]. Flow-mediated dilation quantifies nitric oxide-dependent vasodilatory capacity, which is frequently impaired in hypertensive pregnancy [94,95]. Ex vivo vasoreactivity studies would enable direct assessment of contractile/dilatory responses, allowing structure–function relationships to be established [96]. Absence of functional correlates limits the ability to determine whether structural alterations are translated into functional impairment, compromised vasodilatory reserve, or increased resistance. Future investigations incorporating functional profiling are essential to establishing causative links between PM_2.5_-induced morphological remodeling and cardiovascular dysfunction. Finally, our correlation analyses employed Spearman’s rank correlation without confounding adjustment or multiple comparison correction, which is appropriate for exploratory studies where conservative corrections risk obscuring biological signals [97,98]. The modest sample size (n = 6–7) limits the power of this study for multivariable regression. We report bivariate correlations as descriptive associations identifying potential structure–function relationships that could warrant confirmation in larger studies with multivariate modeling and independent validation [99].

## 5. Conclusions

Pregestational and gestational exposure to wood smoke-derived PM_2.5_ is associated with structural differences in the maternal aortic wall as well as with distinct hemodynamic trajectories during gestation in second-generation Sprague–Dawley rats. These findings suggest that exposure timing is associated with integrated vascular responses characterized by differences in aortic wall organization without evidence of elastic mass loss, as well as by exposure-dependent differences in hemodynamic trajectories during pregnancy. Integration of structural and physiological variables through correlation and principal component analyses show that these changes do not occur in isolation but rather as coordinated vascular–hemodynamic patterns dependent on the temporal window of exposure. Three main contributions emerge from this study. First, it provides experimental evidence consistent with the aortic arch as a potential target of PM_2.5_ exposure derived from biomass combustion, extending current knowledge beyond placental and fetal compartments. Second, the results support the possibility that the pregestational period represents a relevant window of susceptibility, with structural and hemodynamic differences detectable even under reduced gestational exposure conditions, although this requires validation in additional experimental models and human studies. Third, our multivariate approach enabled the identification of coordinated patterns between aortic structure and hemodynamic variables that are not captured by univariate analyses, highlighting the relevance of integrative approaches for evaluating the cardiovascular effects of air pollution during gestation.

## Figures and Tables

**Table 2:** Significant Spearman correlations between aortic wall thickness and hemodynamic parameters across experimental groups. Only statistically significant associations (p<0.05) are presented (n=12 per group). Data are expressed as Spearman’s rank correlation coefficients (*r*) with corresponding two-tailed *p*-values. Positive and negative coefficients indicate direct and inverse monotonic relationships, respectively.

Group	Aortic Layer vs. Hemodynamic Variable	*r*	*p*-Value
FA/FA	Tunica adventitia vs. SBP	0.6014	0.0428 *
FA/NFA	Tunica media vs. SBP	0.7063	0.0129 *
	Tunica adventitia vs. SBP	−0.7273	0.0096 **

Abbreviations: SBP, systolic blood pressure; FA, filtered air; NFA, non-filtered air (PM_2.5_ exposure); tunica intima, innermost layer of the aortic wall; tunica media, middle smooth muscle layer; tunica adventitia, outer connective tissue layer. Statistical significance was set at p<0.05 (* p<0.05; ** p<0.01).

## Data Availability

The original contributions presented in this study are included in the article/Supplementary Materials. Further inquiries can be directed to the corresponding author.

## Supplementary Materials

The datasets generated and analyzed during the current study are publicly available in Zenodo at https://doi.org/10.5281/zenodo.20477816. The repository contains seven supplementary documents that comprehensively support the results presented in this manuscript. Supplementary Document S1 provides aerial and ground-level photographs of the exposure chambers located in the courtyard of the Faculty of Medicine, Universidad de La Frontera, Temuco, along with geographic coordinates and proximity to the Las Encinas air quality monitoring station. Supplementary Document S2 presents descriptive statistics for ambient PM_2.5_, PM_10_, and carbon monoxide (CO) concentrations recorded during the study period and at an annual scale, obtained from the Las Encinas Monitoring Station of the National Air Quality Information System (SINCA). Supplementary Document S3 shows the daily time series of ambient PM_2.5_ concentrations recorded at the Las Encinas Monitoring Station between 1 January and 30 September 2021, including validated, preliminary, and unvalidated records. Supplementary Document S4 contains the complete morphometric dataset for the maternal aortic arch across the four experimental groups, including tunica intima, media, and adventitia thickness; elastic fiber density; and inter-elastic fiber distance, with Kruskal–Wallis and Dunn’s post hoc statistics. Supplementary Document S5 presents maternal hemodynamic parameters, including systolic blood pressure (SBP), diastolic blood pressure (DBP), mean arterial pressure (MAP), and heart rate (HR), measured at gestational days 6, 12, and 18 post-fertilization across all experimental groups. Supplementary Document S6 presents the Rate–Pressure Product (RPP) as an index of myocardial oxygen demand across gestational days 6, 12, and 18 post-fertilization, including group comparisons using one-way ANOVA followed by Holm–Šídák post hoc tests. Supplementary Document S7 provides the complete matrix of Spearman rank correlation coefficients and associated *p*-values between aortic wall thickness parameters and hemodynamic variables at gestational day 18 for each experimental group. All data are openly accessible without restriction and have been deposited to ensure transparency, reproducibility, and reuse of the study findings.

## Abbreviations

The following abbreviations are used in this manuscript:

PM_2.5_	Particulate matter with aerodynamic diameter ≤ 2.5 μm
PM_10_	Particulate matter with aerodynamic diameter ≤ 10 μm
FA	Filtered air
NFA	Non-filtered air
FA/FA	Filtered air during pregestation and gestation
FA/NFA	Filtered air during pregestation and non-filtered air during gestation
NFA/FA	Non-filtered air during pregestation and filtered air during gestation
NFA/NFA	Non-filtered air during pregestation and gestation
KW	Kruskal–Wallis test
H&E	Hematoxylin and eosin
IQR	Interquartile range
CO	Carbon monoxide
NO_2_	Nitrogen dioxide
ROS	Reactive oxygen species
ECM	Extracellular matrix
SMC	Smooth muscle cell
SBP	Systolic blood pressure
HR	Heart rate
PCA	Principal component analysis
G0	Founder generation
G1	First generation
G2	Second generation
ARRIVE	Animal Research: Reporting of In Vivo Experiments
BAM	Beta attenuation monitor
